# Nonlinear Harmonic Distortion of Complementary Golay Codes

**DOI:** 10.1177/01617346221147820

**Published:** 2023-01-12

**Authors:** Fraser Hamilton, Peter Hoskins, George Corner, Zhihong Huang

**Affiliations:** 1 School of Science & Engineering, University of Dundee, Dundee, UK; 2 IMV Imaging (UK) Ltd., UK

**Keywords:** coded excitation, Golay, nonlinear acoustics, simulation

## Abstract

Recent advances in electronics miniaturization have led to the development of low-power, low-cost, point-of-care ultrasound scanners. Low-cost systems employing simple bi-level pulse generation devices need only utilize binary phase modulated coded excitations to significantly improve sensitivity; however the performance of complementary codes in the presence of nonlinear harmonic distortion has not been thoroughly investigated. Through simulation, it was found that nonlinear propagation media with little attenuative properties can significantly deteriorate the Peak Sidelobe Level (PSL) performance of complementary Golay coded pulse compression, resulting in PSL levels of −62 dB using nonlinear acoustics theory contrasted with −198 dB in the linear case. Simulations of 96 complementary pairs revealed that some pairs are more robust to sidelobe degradation from nonlinear harmonic distortion than others, up to a maximum PSL difference of 17 dB between the best and worst performing codes. It is recommended that users consider the effects of nonlinear harmonic distortion when implementing binary phase modulated complementary Golay coded excitations.

## Introduction

Recent developments in the miniaturization of electronic hardware and computer processing have had a profound impact on medical imaging in the 21st century. Although most ultrasound scanners found in a human medical context today are cart-based, handheld and low-power point-of-care ultrasound scanners have the potential to revolutionize the availability of ultrasonic imaging across the world. Key areas of innovation for ultrasound system design include improvements to power consumption and battery life, form factor and portability, and cost.^1,2^

Battery-operated ultrasound scanner design consequently focuses on improving sensitivity without sacrificing efficiency and cost. Advanced imaging techniques requiring high computational load such as Plane Wave imaging or Synthetic Transmit Aperture imaging, or even increasing number of hardware receive channels to increase sensitivity, can have a direct impact on efficiency and cost, therefore other methods of improving sensitivity should be examined.

Pulse compression has been used in a wide range of technologies from it’s conception in radar imaging through to telecommunications and ultrasonic imaging, which first saw successful implementation in diagnostic ultrasound machines in the 1990s^3^ and grew in popularity as ultrasound scanners reached the Mechanical Index (MI) limit imposed by regulatory bodies.

Pulse compression using binary phase coded excitations are of particular interest to low-power, low-cost ultrasound scanner design as binary codes only require simple bi-level pulse generation architectures, rather than LFM “chirp” imaging which requires costly and complex multilevel pulser hardware.^4^ One binary phase coded excitation scheme of note uses the Golay complementary sequences, consisting a pair of equal length codes with autocorrelation functions which sum to produce a central peak of 2N, where N is the length of the code in positive and negative symbols, and zero range side-lobes.^5^ Golay complementary codes require two transmit-receive cycles to obtain a single line of sight, meaning non-stationary features can corrupt the complementary summation, leading many to discard them.^4^ Despite this, some have found their use in blood flow imaging^6,7^ and others have devised motion compensation techniques to ensure good summation.^3^

Every aspect of pulse compression using binary phase coded excitations must therefore be optimized to gain the most performance for low-power, low-cost ultrasound systems.

There has, to date, been very little research into the interaction of complementary Golay codes with nonlinear acoustics theory. A seminal paper covering pulse compression for ultrasonic imaging reviewed the use of both Golay and Chirp coded excitations in detail for ultrasound scanners.^4^ Although an extremely informative paper, issues surrounding nonlinear propagation and attenuation were only briefly investigated. Simulations of Golay codes propagating through linear and nonlinear media, with B/A of 0 and 8 respectively, were compared across a range of attenuation values and authors found that, although the sidelobes of binary phase codes increase due to harmonic distortion, sidelobe levels reduced as attenuation increased. This indicated that frequency dependent attenuation was aiding the reduction of harmonics in Golay codes, thus maintaining satisfactory complementary summation.

However, ultrasound in medical settings rarely travels through homogenous tissue. Anatomical heterogeneities present propagation media of varying acoustic properties, for example, the skin layer, fat, muscle, and fluid-filled regions as in obstetric imaging. As such, the degree of attenuation cannot be guaranteed.

Therefore the robustness of Golay sidelobe cancelation in nonlinear propagation media can be better understood by first ignoring attenuative effects and considering in isolation the impact of nonlinear harmonic distortion.

This paper will examine in simulation the impact of nonlinear harmonic distortion on the sidelobe cancelation performance of complementary Golay codes. Some background knowledge pertinent to this article will be presented, followed by a description of the model used to simulate acoustic propagation. Harmonic distortion of Golay codes will be investigated and compared using Peak Sidelobe Level (PSL), followed by an analysis of 96 simulated complementary sequences.

## Pulse Compression

Sets of binary codes exist which are suitable for constructing coded excitations, whereby the symbols of each code can be represented by +1 or −1 . Rather than direct transmission, the accepted method of implementing binary coded excitation in ultrasound imaging is to modulate a base sinusoidal pulse train of length N cycles with 0 to π phase shifts to represent +1 and −1 code symbols.^4^

Two commonly used sets of binary sequences suitable for pulse compression in ultrasound are Barker sequences and Golay complementary sequences. For the purposes of this research, we will consider only Golay complementary sequences.

### Golay Coded Excitation

Transmission, decode, and summation steps of the binary complementary series devised by Golay^5^ are well described in literature.^4,8^

The convolution stage of pulse compression is implemented as a matched filter, with the template constructed via the time-reversal of the transmitted coded excitation. Note that the matched filter result generates peaks at points of partial correlation symmetrically around the mainlobe. These sidelobes are an artifact of the correlation process and can appear as weak peaks on either side of the mainlobe after envelope detection.

If not adequately minimized, the axial resolution of a system using pulse compression imaging will be significantly reduced: high amplitude sidelobes would mask mainlobe echoes from nearby weakly echogenic, and potentially significant, targets.^9^ The aim of good pulse compression implementation, therefore, is to transmit sequences which give a mainlobe at the point of maximum correlation and sidelobes close to zero at all other points.

The final stage of pulse compression using Golay codes is complementary summation, whereby the mainlobes are reinforced to provide a single peak at 2N and sidelobes are canceled as a result of destructive interference. Note that the sidelobes must be identical, albeit inverted, to provide perfect cancelation to 0.

### Impact of Nonlinearity on Pulse Compression

Nonlinear variations between pressure and density in propagation media cause waveform compressional and rarefactional pressure components to travel at different speeds, altering the amplitude, shape, and spectra of the propagating waveform. The peak compression components of the waveform accelerate toward π / 2 π/2 , whereas the peak rarefaction components decelerate to approach π / 2.^10^ This is reflected in the spectra of the waveform as upper harmonics of the fundamental increase.

The decoding matched filter extracts a known signal which has been distorted by some linear process, such as additive white noise, however the harmonic distortion created by nonlinearities nonlinear propagation media will logically cause the receiving matched filter template to be suboptimal, as the positive and negative portions of the waveform travel toward and away from the zero crossing, respectively.

Various metrics have been proposed as indicators of nonlinearity, and, although no single metric is best placed to represent all facets of nonlinear propagation,^11^ the Spectral Index ( SI ) has the benefit of representing harmonic distortion across the whole frequency spectrum and is agnostic of pulse shape and frequency, source pressure, and material properties^12^:

(1) $$SI=\frac{\int_{f_{a}}^{\infty }P_{f}df}{\int_{0}^{\infty }P_{f}df}$$

Where $f_{a}$ is arbitrarily chosen at $1.5f_{c}$ , where $f_{c}$ is the fundamental, and $P_{f}$ is the power of the frequency spectrum. This metric will be used to represent the degree of harmonic distortion present in the following simulations.

## K-Wave Simulation

The k-Wave toolbox for MATLAB can be used to assess nonlinearity through different media. The tool iteratively solves coupled first-order acoustic equations in one, two, and three dimensions, a more efficient method than solving the equations using finite element methods. For a detailed description and experimental validation of the *k*-Wave toolbox, see Treeby and Cox,^13^ Treeby et al.,^14^ and Martin et al.^15^

The simulation is designed as a 2D 40mm × 40mm body of water at $20^{\circ }C$ (properties listed in Table 1) with an arc acoustic source object representing a concave element round transducer of 18mm diameter and 75mm radius. 2048 × 2048 grid points represent the simulation space, giving a grid spacing of 19µm 19μm , including a PML of 40 × 40 grid points. The maximum spatial frequency supported by the grid is 37MHz . A binary sensor mask is used to place 100 sensors in the axial midpoint of the propagation medium at steps of 0.4mm per sensor, thus mimicking a hydrophone moving in-step with wave propagation. This approach reduces simulation complexity, as compared to placing reflecting scatterers within the medium, since only the interaction of transmitted Golay codes with nonlinear harmonic distortion is investigated. The CFL number is 0.3 , resulting in a temporal sampling frequency of 248MHz which is far greater than the maximum reproducible spatial frequency, but necessary to ensure stable simulations.

**Table 1.** Material Properties of Water @ 20ºC.

ρ $\left(kg/m^{3}\right)$	c (m/s)	B/A	$\alpha_{0}$ $\left(dB/MHz^{y}-cm\right)$	y
998	1482.8	4.96	2.17e-3	2.00

**Figure 1.**
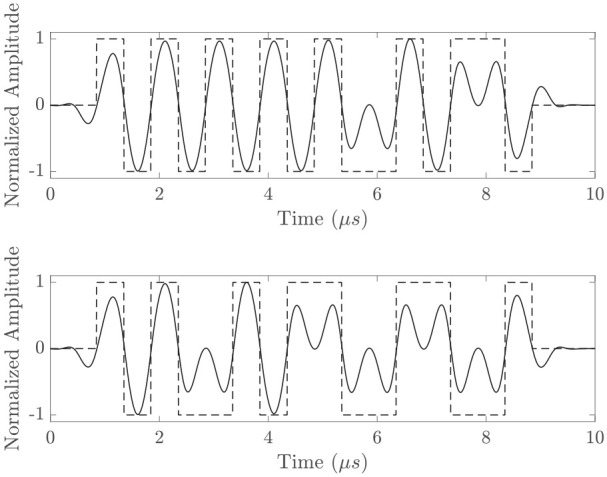
Phase-modulated 1 MHz square wave codes (dashed) and transmitted result after 85% fractional bandwidth bandpass filter (solid) for Golay (a) and (b) top and bottom respectively.

The input source signal is designed to mimic the limited bandwidth of a typical ultrasound transducer. Transmissions are first defined as a square wave pulse train to imitate the behavior of bi-level pulsers used in conventional ultrasound systems and modulated according to Golay codes, for example, given in Table 2 generated by the MATLAB code presented in Trots et al.^8^ Transmissions are then filtered through a 512-tap bandpass FIR filter with center frequency of 1 MHz and 85% fractional bandwidth, resulting waveforms are given in Figure 1. A final non-causal filtering step is included, as recommended by the *k*-Wave documentation to eliminate high frequencies from the transmit signal that are not supported by the grid, before the input signal is amplified by a source pressure of 3MPa . Although most diagnostic ultrasound source pressures fall below this level, 3MPa was required to generate harmonic distortion with the above stated simulation space and grid point density.

**Table 2.** Complementary Symbols for Code A and Code B.

Golay A	+	+	+	+	+	−	−	+
Golay B	+	+	−	−	+	−	+	−

The B/A parameter of nonlinearity can be omitted when defining the medium properties in *k*-Wave to allow investigation only of linear propagation. Simulations of complementary pairs are conducted for both nonlinear and linear cases, represented in all plots by a solid line and dashed line respectively.

Simulations are performed using the optimized *C*++ program, providing an order of magnitude speed increase when run on an NVIDIA CUDA-enabled GPU. The following simulations are performed on a Windows Server with an Intel Xeon Silver 4214 CPU @ 2.20 GHz processor, 32 GB RAM, and an NVIDIA Quadro P2000 GPU with 5 GB GDDR5 memory.

Decoding is performed with a matched filter using the time-reversal of the square wave signals shown in dashed lines in Figure 1 as the template, with number of coefficients equal to the length of the transmitted pulse at the temporal sampling rate.

Sidelobe cancelation performance is quantified by the Peak Sidelobe Level (PSL), defined as the ratio of the peak mainlobe to that of the peak sidelobe in decibels, given in (2):

(2) $$PSL=20log10\frac{max(A_{mainlobe})}{max(A_{sidelobe})}$$

## Results

Consistent with both the theory and simulation of a conventional sinusoid, Golay waveforms prior to decoding become distorted in both the peak compression and rarefaction phases throughout the propagation distance.

The differences between the time-domain view of linear and nonlinear codes are not as prominent after decoding, proving the effectiveness of the matched filter.

Table 3 compares the SI of the conventional sinusoid and Golay codes. Spectral energy transferred from the fundamental of the coded excitations follows the same trend as that of the conventional sinusoid. SI of all waveforms are below 25% for 20 mm of the 40 mm simulation space, then rapidly increase at a rate of at least 2% per mm until the maximum recorded point at 36 mm, indicating that the greatest waveform distortion occurs in the latter half of the simulation. Further, the SI of the complementary sum is notably lower than that of the sinusoid or coded excitations.

**Table 3.** Spectral Index (%) of Golay Codes Given in Table 2 Prior to Decoding and After Complementary Summation, Compared with Conventional Sinusoid, for Increasing Distance Z Z From the Transducer Face. Also Shown are the PSL Values for Before and After the Mainlobe, Denoted “Pre” and “Post.”.

Z (mm)	*SI* (%)				PSL (dB)	
	Sinusoid	Code A	Code B	Sum	Pre	Post
0	6.15	9.04	14.42	2.28	−198	−148
4	7.98	10.48	16.16	2.98	−118	−120
8	10.97	12.58	16.95	2.92	−104	−104
12	17.24	17.88	21.58	3.86	−94	−97
16	14.56	15.79	20.67	3.25	−88	−93
20	22.37	20.72	24.07	3.81	−85	−87
24	33.70	30.35	32.14	5.95	−81	−81
28	45.34	40.98	41.80	8.63	−78	−76
32	56.88	52.07	51.96	11.47	−76	−72
36	65.90	61.58	60.98	14.10	−75	−70

The evolution of sidelobes with increasing SI for the nonlinear case can also be seen in Table 3, where “Pre” and “Post” denote the sidelobe levels on either side of the mainlobe of the complementary sum. PSL rises from −148dB at the transducer face to −70dB at Z=36mm whereas the sidelobes for the linear case, not shown in Table 3, peak at −196dB .

PSL values nearest to and furthest from the transducer face for values of Z in the nonlinear case are given in Table 3, which rises logarithmically from −198dB at the transducer face then rapidly to a knee-point of −94dB at Z=16mm before leveling off to approach −70dB at Z=36mm . The linear PSL, not shown, consistently hovers around −196dB for the duration of the propagation.

**Figure 2.**
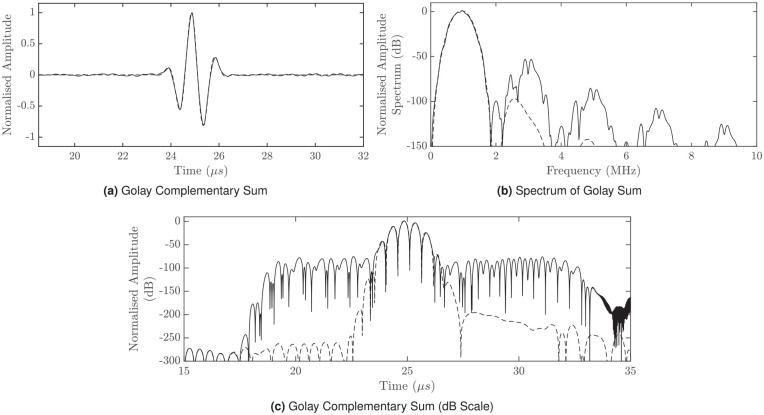
Complementary summation of decoded Golay codes recorded at 28 mm from the transducer face with nonlinear and linear cases represented by the solid and dashed lines respectively. 2(a) shows the final pulse compressed waveform, with notable sidelobes on either side of the mainlobe. 2(b) shows the corresponding spectrum. 2(c) shows a dB plot of the complementary summation waveform, where a significant difference can be seen between the nonlinear and linear cases. (a) Golay Complementary Sum, (b) Spectrum of Golay Sum, and (c) Golay Complementary Sum (dB Scale).

Normalized and dB plots of the complementary sum at z=28mm are shown in Figure 2, with nonlinear and linear cases depicted by the solid and dashed lines respectively. Whilst the mainlobe of the complementary sum is similar in both cases, the sidelobes of the nonlinear simulation are significantly higher than the linear case, as seen by the dB plot.

As seen in Table 3, the notably lower SI of the complementary sum compared to conventional sinusoid is reflected in Figure 2(b), where the upper harmonic levels of the nonlinear case are considerably lower than the fundamental. Note that the spectra after decoding preserves the odd-numbered harmonics.

Simulations are repeated with a range of 96 different algorithmically-generated complementary pairs^8^ to assess their relative performance.

**Figure 3.**
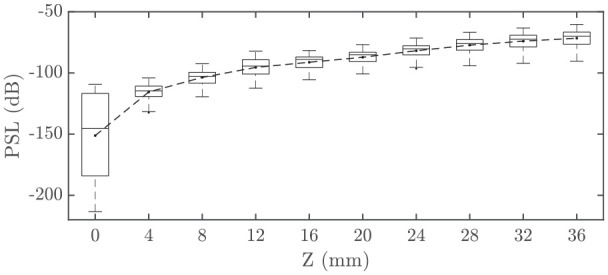
Box plots of PSL against Z for 96 Golay complementary pairs for the nonlinear case, with mean PSL result depicted by the dashed line.

Box plots of PSL against Z for 96 code pairs are shown in Figure 3 with the mean value represented by the dashed line. PSL values after the mainlobe are not shown here but follow a trend similar to that shown in Table 3. Results were obtained for the linear case, where the PSLs for all simulations were consistently below −196dB .

Sidelobe levels are widely distributed at the transducer face, however the inter-quartile range rapidly narrows at Z=12mm and above, indicating that all simulated codes degrade with nonlinear harmonic distortion. For Z=36mm , maximum and minimum ranges are −70dB and −94dB respectively, proving that some codes present a higher PSL than others under harmonic distortion. Both mean and median PSL values increase logarithmically with Z .

It is well known that Golay swap sets exist, whereby a Golay code can be inverted and/or reversed to yield a new complementary pair. Two such swap sets were identified within the 96 simulated codes, notated hereafter for brevity in hexadecimal, and given in Table 4. Codes within the swap set exhibit different PSL values in the presence of nonlinearity. The nonlinear case shows significant variation on either side of the mainlobe. By selecting Code B 47 instead of E2 , for instance, the pre-mainlobe PSL degrades by 16dB whereas the post-mainlobe PSL improves by 13dB . All linear case PSLs were found to be consistently below −110dB . Different swap sets were also identified and results logged, which showed comparable results.

**Table 4.** Comparison of PSLs Before and After the Mainlobe for Golay Swap Sets Notated in Hexadecimal Recorded at Z = 36mm, Denoted as “Pre” and “Post” Respectively.

Code A	Code B	PSL Pre (dB)	PSL Post (dB)
ED	E2	−84	−63
ED	B8	−78	−66
ED	47	−68	−76
ED	1D	−74	−65
BE	B1	−79	−68
BE	8D	−63	−76
BE	72	−66	−67
BE	4E	−62	−67

**Figure 4.**
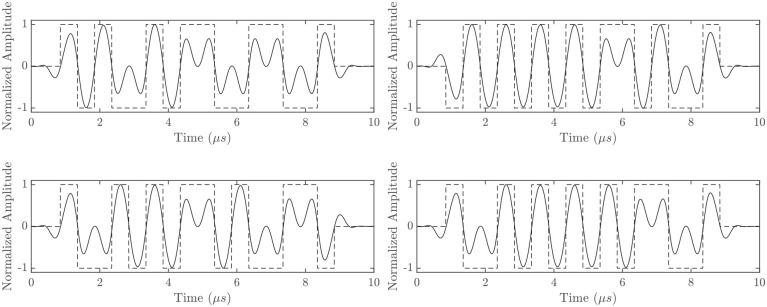
Source waveforms Code *A* = CA and Code *B* = 06 (top left & top right respectively) and Code *A* = *8D* and Code *B* = 82 (bottom left & bottom right respectively). Where the source waveform and square wave pulse trains are represented by the solid and dashed lines respectively.

Complementary pairs were sorted in order of their PSL performance for both before and after the mainlobe at Z=36mm . The best and worst performing codes are given in Table 5, again in hexadecimal notation, with corresponding source transmission waveforms in Figure 4. For pair A=8D & B=82 , the post-mainlobe PSL was −62 dB for the nonlinear case, compared to −198 dB for the linear case. Linear PSLs for both pairs in Table 5 were consistently below –150 *dB.*

**Table 5.** Code Pairs Notated in Hexadecimal with Highest and Lowest PSLs Before and After the Mainlobe Recorded at Z = 36mm, Denoted as “Pre” and “Post” Respectively.

		PSL (dB)	
Code A	Code B	Pre	Post
CA	06	−75	−79
8D	82	−66	−62

## Discussion

Simulations presented here reveal that sidelobe levels of complementary pairs deteriorate in the presence of nonlinear harmonic distortion, with PSLs found to be significantly higher in the nonlinear case than that of the linear case. Previous work determined that this would have little effect in materials with high frequency dependent attenuation properties, however it must be acknowledged that this is not the case in all materials, be it anatomical or otherwise.

Significant harmonic distortion was observed throughout the propagation of phase modulated Golay codes in simulation. Although decoding led to a marked reduction in harmonic content, final complementary summation revealed significant sidelobe levels, indicating that the matched filter using the template of an ideal code was not optimal. PSL rose from, on average, −151dB at the transducer face to −71dB at Z=36mm prior to the mainlobe.

Although the average PSL of −71dB relative to the mainlobe is likely acceptable for 12-bit ADC architectures, sidelobes of some complementary pairs registering as high as −62dB would degrade interpretation of targets. Further, sidelobes would be more apparent on newer architectures built around 14-bit ADC devices with dynamic ranges of −84dB relative to full scale.

An investigation into 96 complementary pairs revealed that some codes perform significantly better than others. A trade-off clearly exists between PSL performance on either side of the mainlobe. Golay swap sets exhibited such behavior, with code pairs within a swap set giving better PSL performance prior to the mainlobe rather than afterward. Of all 96 simulated codes, the codes with the lowest and highest combined PSLs at Z=36mm , that is, the best and worst performers, were A=CA , B=06 and A=8D , B=82 , respectively.

The reason why some complementary pairs result in lower PSLs than others is understandably of significant importance. The number of +1 to −1 transitions were investigated, as were the number of positive and negative symbols, and whether codes began with +1 or −1 symbols—none of which provided definitive answers. Further, the logarithmic increase of PSL seen in Figure 3 does not follow the same trend line as that of the SI for either code transmissions or the summed result. The SI of coded excitations presented in Table 3 increase faster than that of the conventional sinusoid in the first 16mm of propagation, indicating that energy is more readily transferred to higher harmonics. Note, however that the spectrum of a linear Golay code contains higher harmonics than that of the conventional sinusoid due to the complex wave shape.

Notably the level of all even harmonics is far reduced after decoding due to the convolution theorem. The frequency response of each matched filter exhibits stopbands at even harmonics with complex passbands at odd harmonics. Further investigation into the interaction of this complicated frequency response with nonlinear Golay waveforms is clearly valuable but outwith the scope of this paper.

The asymmetry metric $p_{c}/p_{r}$ ^12^ presents the amount of distortion present in a waveform as the ratio of the peak compressional component to that of peak rarefaction. This was investigated for the coded excitations and found to follow a similar trend to that of SI , so does not indicate a correlation between asymmetry and PSL.

The above results confirm that harmonic distortion can negatively impact the PSL performance of complementary Golay codes, but note the ideal conditions used to generate high SI.

The acoustic shock equation^12^:

(3) $$\sigma =\frac{\beta p_{0}2\pi f_{0}z}{\rho_{0}c_{0}^{3}}$$

dictates that the formation of shock is proportional to the material coefficient of nonlinearity β, transmitted source pressure $p_{0}$ , source frequency $f_{0}$ , and distance from the transmitter z .

The source pressure of 3MPa used in the above simulations is higher than that used in diagnostic medical ultrasound imaging, thus simulations from Figure 1 were repeated with lower transmitted pressures of 0.8MPa . This resulted in a Golay sum at z=36mm with SI of 3.26% and a peak PSL of –97 dB. Note that the effects of frequency dependent attenuation in tissue would further attenuate upper harmonics thus reducing PSL.^4^

It is therefore unlikely that complementary Golay codes would develop high sidelobe levels in medical diagnostic ultrasound imaging as a result of nonlinear harmonic distortion.

Although the results presented here are only a result of simulation data, it is worth noting that the *k*-Wave simulation toolbox has been verified as accurate with experimental data and that the number of observations presented here are easier obtained in simulation than experimentally. Further, the above simulations make use of “turning off” nonlinear acoustics physics which is of course not possible experimentally.

## Conclusion

The goal of this article was to examine sidelobe degradation of complementary Golay coded excitations in the presence of nonlinear harmonic distortion, which was achieved by simulating the propagation of the complementary transmissions in water with both linear and nonlinear acoustics theory using the *k*-Wave toolbox for MATLAB.

Previous authors have shown that frequency dependent attenuation in homogenous media can sufficiently attenuate harmonic distortion of complementary codes, thus maintaining satisfactory summation.^4^ However, the simulation results presented here confirm that propagation through water, and therefore other materials with very little frequency dependent attenuation properties, can lead to increased sidelobe levels. Whilst the harmonic distortion observed in this paper originates from the nonlinear relationship between pressure and density in tissue and fluids, the results are applicable regardless of the source of nonlinearity; distortion may arise from multiple sources in the imaging chain between signal generation to matched filter reception.

These findings demonstrate that sidelobe performance of complementary Golay codes will degrade in the presence of nonlinear harmonic distortion, but that the safety limits on medical diagnostic ultrasound imaging will prevent such artifacts from appearing in clinical B-Mode imaging. These findings may, however, have implications for other areas of ultrasonic systems, such as NDT.

It is recommended that system designers aiming to extract the maximum performance of ultrasound devices using pulse compression with complementary codes must consider that nonlinear harmonic distortion can degrade PSL performance, and that proper selection of Golay codes which give desirable cancelation properties should be used.

Readers should also be aware that these results have implications for not only ultrasonic devices in nonlinear propagation media, but to other technologies where sources of nonlinearity can distort transmitted complementary codes.

Future research can build on these observations with simulations approximating anatomical structures with varying nonlinear materials, experimental validation with hydrophones, and also determining why some complementary Golay codes perform better in the presence of nonlinear harmonic distortion than others.
